# Ets-2 and p53 mediate cAMP-induced MMP-2 expression, activity and trophoblast invasion

**DOI:** 10.1186/1477-7827-7-135

**Published:** 2009-11-25

**Authors:** Elsebeth Staun-Ram, Shlomit Goldman, Eliezer Shalev

**Affiliations:** 1 Laboratory for Research in Reproductive Sciences, Department of Obstetrics and Gynecology, Ha'Emek Medical Center, Afula, Israel; 2 Rappaport Faculty of Medicine, Technion-Israel Institute of Technology, Haifa, Israel

## Abstract

**Background:**

We have previously shown that Matrix metalloproteinase (MMP) -2 is a key-enzyme in early trophoblast invasion and that Protein Kinase A (PKA) increases MMP-2 expression and trophoblast invasion. The aim of this study was to examine MMP -2 regulation by PKA in invasive trophoblasts: JAR choriocarcinoma cell-line and 6-8 w first trimester trophoblasts.

**Methods:**

The effect of Forskolin (PKA) on MMP-2 expression was assessed by Northern Blot and RT-PCR. Possible transcription factors binding to consensus MMP-2 promoter sequences in response to Forskolin, were detected by EMSA binding assay and their expression assessed by western blot analysis. Antisense transfection of relevant transcription factors was performed and the inhibitory effect assessed on MMP-2 expression (RT-PCR), secretion (zymography) and trophoblast invasiveness (transwell migration assay).

**Results:**

We found that Forskolin increased MMP-2 mRNA in JAR cells within 24 hours, and induced binding to p53, Ets, C/EBP and AP-2. Transcription factors Ets-2, phospho- p53, C/EBP epsilon, C/EBP lambda and AP-2 alpha bound to their respective binding sequences in response to Forskolin and the expressions of these transcription factors were all elevated in Forskolin- treated cells. Inhibition of Ets-2 and p53 reduced MMP-2 expression, secretion and invasiveness of Forskolin treated cells.

**Conclusion:**

MMP-2 is regulated by PKA through several binding sites and transcription factors including Ets-2, p53, C/EBP, C/EBP lambda and AP-2 alpha. Ets-2 and p53 mediate cAMP- induced trophoblast invasiveness, through regulation of MMP-2.

## Background

During implantation the trophoblast exhibit behavior similar to tumor cells, including tissue invasiveness, metastasis, loss of contact inhibition, escape from immune surveillance and massive proliferation ability [1]. However, in sharp contrast to aggressive tumors, trophoblast invasion process is a spatial and temporal strictly regulated process, controlled by multiple interactions between cells and environment, mediated by several factors including growth factors, hormones and cytokines [2,3].

Trophoblast are highly invasive due to the secretion of extracellular proteases, like Matrix Metalloproteinases (MMPs), mediating degradation of the extracellular matrix (ECM), and of their balancing inhibitors Tissue inhibitors of Metalloproteinases (TIMPs) [4-6]. The two gelatinases, MMP-2 and MMP-9, digest type IV collagen, the main component of the basement membrane and are therefore regarded as key enzymes in trophoblast invasion [5,7-9]. MMP-2 and MMP-9 expression is differential during the first trimester of pregnancy. MMP-2 production is dominant until 8 weeks of gestation and then declining, whereas MMP-9 production significantly increases from 8 weeks, causing a shift of dominant gelatinase from MMP-2 to MMP-9 from 9 week of gestation [8,9]. The implantation process occurs by two waves of invasion by the trophoblast, the first strictly controlled to the implantation site and completed at 8 weeks of gestation, and the second wave deep into the uterine wall at the end of first trimester [10,11]. MMP-2 is likely to be the key-enzyme in the first physiological invasion wave, whereas MMP-9 may be the key-enzyme in the second invasion wave.

The JAR cell line, obtained from choriocarcinoma, is comprised of highly invasive trophoblast, expressing mainly MMP-2 and to a much lesser extent MMP-9 [8,12,13]. JAR cells were shown in our lab to resemble early 1^st ^trimester trophoblast in gelatinase profile and invasiveness, and can therefore be used as a good model for studying early invasive trophoblast regulation [8]. Several factors with importance in embryo implantation act via cyclic adenosine monophosphate (cAMP)-protein kinase A (PKA) signal transduction pathway, including human chorion gonadotropin (hCG), the primary signal of an implanting pregnancy [14,15]. hCG was found to increase collagenolytic activity, trophoblast invasion and migration [15-17], and MMP-9 [18]. Forskolin is a prototypical stimulator of the cAMP pathway by direct activation of adenylate cyclase [19]. In a previous study, we showed that Forskolin (PKA) induces MMP-2 and MMP-9 and trophoblast invasiveness in both JAR cells and 1^st ^trimester trophoblasts [8]. Forskolin has been used also in other studies to induce invasion or invasion-related molecules [19,20]. Additionally, Forskolin/cAMP has been used to induce syncytiotrophoblast formation [21,22]. The use of cell-lines of different origin as well as primary or term trophoblast may be the cause of these differential effects.

Most MMPs are regulated at the transcriptional level, by several cytokines and growth factors, which influence multiple signaling pathways [23]. The MMP gene promoter contains several *cis*-regulatory elements, often acting synergistically, with varying importance and effect, depending upon cell-type and inducer [6]. The MMP -2 promoter lacks a typical TATA-box, but is not refractory to modulations as previously thought [24,25]. The transcriptional regulation on MMP-2 in trophoblasts is not yet clear. The aim of this study was to investigate the transcriptional regulation of MMP-2 in cAMP- activated invasive trophoblast. Other MMP regulation levels include mRNA stabilization, protein activation or inhibition, surface localization and secretion; however these levels were not part of the present study.

## Methods

### Cell culture

The JAR (Jar, HTB 144, ATCC) human choriocarcinoma line was a generous gift from Pr. Hochberg, Hebrew University, Israel. JAR cells were cultured in M-199 medium containing 10% Fetal Calf Serum (FCS) and penicillin/streptomycin (all Beit-Ha'Emek, Israel) at 37°C in a humidified, 5% CO_2 _atmosphere. After 24 h, medium was changes to M-199 medium with 1.5% serum, in the presence or absence of Forskolin 10 μM (Sigma, ST Louis, MO, USA).

### Isolation and cultivation of human cytotrophoblast

Human trophoblast cells were obtained from legal abortions (6 to 8 weeks), with the approval of the local ethical committee (in compliance with the Helsinki Declaration) and the consent of the participating patients. Trophoblast cells were isolated according to a protocol generously provided by Desoye G, clinic of Obstetrics and Gynecology, Austria. Briefly, placenta were washed in saline, then the minced trophoblastic villi were digested by 0.25% trypsin and DNase I (both Sigma, ST Louis, MO, USA), and CTB separated from blood cells and decidua on a discontinuous Percoll gradient (Sigma, ST Louis, MO, USA). Contaminating

leukocytes were removed by immunopurification with anti- CD45RB (DAKO, Glostrup, Denmark) coupled to magnetic particle. This method supplies a 95-98% purity of trophoblast, including all trophoblast sub-groups [26]. Cells were cultured in M-199 medium supplemented with 1.5% FCS and 1% penicillin/streptomycin for the various experiments.

### Substrate-gel-electrophoresis (zymography)

Proteolytic activity of culture supernatants were detected using gelatin-substrate gel electrophoresis (zymography). Samples, normalized to cell count performed with XTT Reagent kit (Beit-Ha'Emek, Israel), were diluted in sodium dodecyl sulphate (SDS) sample buffer and electrophoresed through a 10% SDS-polyacrylamid gel containing 0.5% gelatin. Afterwards, gels were rinsed in 2.5% Triton X-100 (Sigma, St. Louis, MO, USA) and incubated for 24 h at 37°C in 0.2 mol/l NaCl, 5 mmol/l CaCl_2_, 0.2% Brij 35 and 50 mmol/l Tris, pH 7.5. Gels were stained with Coomassie Blue G in 30% methanol and 10% acetic acid. Each gelatinase band was identified in accordance to the molecular weight and commercial standards (data not shown). Bands on each gel were compared to their control band and quantified with a densitometer system, endowed with Bio-Capt and TINA software (Raytest, Staubenhardt, Germany).

### Matrigel invasion assay

Matrigel (1 mg/ml) (BD Biosciences, Franklin Lakes, NJ, USA) diluted in serum free media was added to upper chamber of 24-well transwell plate (8 μm pores, Corning, MA, USA). 10^5 ^cells in 100 μl media were added to the upper chamber, and 500 μl media added to the lower chamber. 10 μM Forskolin was added to medium in upper and lower wells and cells incubated at 37°C for 48-72 hours. Cells (10^5^/well) seeded simultaneously in the same media in a well without transwell, served as reference of total seeded cells. After incubation non-invaded cells on top of the transwell were scraped off with a cotton swab and the amount of invaded cells in the lower well as well as the amount of total seeded cells was evaluated with XTT Reagent kit. The percent of invasion was calculated as:

Invasion was expressed as Invasion Index (Percent of control).

### RNA extraction

Total cellular RNA was extracted using a total RNA isolation kit EZ-RNA (Beit HaEmek, Israel) according to manufacturer's instructions. RNA concentration was determined spectro-photo-metrically.

### Northern blot analysis

15 μg of total RNA was electrophoresed on a 1% denaturating agarose gel and transferred to a nitrocellulose membrane. The membrane was hybridized with 2 μg/lane complimentary digoxigenin-labeled DNA probe for MMP-2 or GAPDH (Biognostik, Göttingen, Germany) using the digoxigenin (DIG) Easy Hyb system, according to manufacturer's instructions (Roche, Basel, Switzerland). The probes were previously labeled with digoxigenin by terminal transferase labeling kit (Roche, Basel, Switzerland). The membrane was then rinsed in 2× SSC/SDS and in 0.1× SSC/SDS. Bands were detected with anti-digoxigenin-AP antibody and the chemiluminescent substrate CSPD (both Roche, Basel, Switzerland) on X-ray film.

### Reverse transcription-polymerase chain reaction (RT-PCR)

First-strand cDNA was synthesized from 2-4 μg of total RNA using 0.5 μg oligo (dt) primer, 1 mM dNTP, 15 Units AMV reverse transcriptase (Promega, Mannheim, Germany), 5 mM MgCl_2 _and buffer (10 mM Tris0Hcl, 50 mM KCL, o.1% Triton x-100). RT-PCR was performed in a final volume of 50 μl containing 10 μl cDNA, 10 mM dNTPs (Promega, Mannheim, Germany), 1.5 U Taq polymerase (Sigma, ST Louis, MO, USA), 50 pmol of each primer and 1× reaction buffer. Amplification parameters were 30 cycles as follows: denaturation, 30 sec at 94 °C, annealing, 60 sec at 58 °C, extension, 90 sec at 72 °C. MMP-2 primer forward (F): ACCTGGATGCCGTCGTGGAC and reverse (R): TGTGGCAGCACCAGGGCAGC, GAPDH primer F: TGAACGGGAAGCTCACTGG and R: TCCACCACCCTGTTGCTGTA (IDT Inc., Hy-Labs, Rehovot, Israel). Amplification of GAPDH gene transcripts was performed simultaneously to confirm RNA integrity, efficiency and for quantification of cDNA. Negative control reactions containing samples without cDNA or Taq enzyme were used (data not shown). RT-PCR products were analyzed by 2.5% agarose gel electrophoresis. Images were captured with Polaroid (Hertfordshire, UK) film under UV light. Products were quantified using a Densitometer system endowed with TINA software (Raytest, Staubenhardt, Germany).

### Preparation of nuclear extract

JAR cells or 1^st ^trimester trophoblasts were incubated in serum-free medium for 24 hours, with or without 10 μM Forskolin. Cells were lysed with swelling buffer (10 mM Hepes, 10 mM KCL, 1 mM EDTA, 1 mM DTT, 1 mM PMSF, 10 μg/μl Leupeptin, 50 μg/ml Aprotinin) for 15 min at 4°C, then 10% NP-40 was added for lysis and cells centrifuged at 4°C for 3 min at 3000 RPM. The cytosol protein extract was collected and the remaining pellet resuspended in extraction buffer (20 mM Hepes, 200 mM NaCl, 1 mM EDTA, 1 mM DTT, 1 mM PMSF, 10 μg/μl Leupeptin, 50 μg/ml Aprotinin), vortexed and kept at 4°C for 15 min, then centrifuged at 4°C for 5 min at 12000 RPM. The protein concentration was determined with Bradford reagent assay (Bio-Rad laboratories, Washington, DC). Nuclear extract was assayed for the presence of transcription factors, entering the nucleus in response to stimulus.

### Electrophoretic mobility shift assay

Transcription factor activities were assessed by EMSA using double-stranded oligonucleotide corresponding to the consensus sequences of the MMP-2 promoter [25]. Oligonucleotides (Activator protein (AP)-1, AP-2, selective promoter factor (SP)-1, erythroblastosis virus E26 oncogene homolog (ETS), CCAAT-enhancer-binding protein (C/EBP), cAMP regulatory element binder (CREB), protein 53 (p53) [Santa Cruz Biotech, California, USA, (Table 1)] were labeled with digoxigenin (Roche, Basel, Switzerland) using Terminal Transferase according to manufacture's instructions in dig-labeling kit (Roche, Basel, Switzerland). Binding reactions were conducted by incubation of 6 μg of nuclear extract from JAR cells with digoxigenin- labeled oligonucleotide probes at 30°C for 30 min. in binding buffer containing 15 mM Hepes, 90 mM KCL, 6% glycerol, 3 mM DTT, 0.5 μg Poly [d(I-C)] and 0.4 ng oligonucleotide. Subsequently, DNA-protein complexes were separated from unbound oligonucleotides on a pre-electrophorized 6% polyacrylamide gel and electrotransferred to positively charged nylon membranes (Roche, Dyn Diagnostics Israel). The DNA-protein complexes were fixed by baking 30 min at 120°C. Bands were detected with anti-digoxigenin-AP antibody and the chemiluminescent substrate CSPD (both Roche, Basel, Switzerland) on X-ray film. Sequence specificity of nuclear protein-oligonucleotide interaction was confirmed by competition with cold oligonucleotide and by the addition of 100 μm PKA inhibitor H89 (Sigma, St. Louis, MO, USA) to the culture media. For supershift detection, 1 μl antibody (200 μg/0.1 ml) was added to nuclear extract mix and incubated for 30 min at 4°C before incubation with oligonucleotides, and a 4.5% polyacrylamide gel was used for separation. Antibodies used for supershift are listed under western blot.

**Table 1:** Commercial consensus oligonucleotide sequences (Santa Cruz)

Oligonucleotide	**Catalog No**.	Consensus binding motif
Ap-1	Sc-2501	5' TGACTCA 3'
Ap-2	Sc-2513	5' GCCCGCGG 3'
C/EBP	Sc-2525	5' TTGCGCAA 3'
p53	Sc-2579	5' GAACATGTCTAAGCATGCTG 3'
CREB	Sc-2504	5' TGACGTCA 3'
SP-1	Sc-2502	5' GGGGCGGGGG 3'
Ets	Sc-2555	5' CAGGAAGT 3'
NFkB	Sc-2505	5' GGGGACTTTCCC 3'

### Western blot analysis

Equal amounts of nuclear (according to Bradford assay) and molecular mass marker were denatured and subjected to a 10% SDS-polyacrylamide gel electrophoresis. Proteins were electrotransferred to 0.45 μm nitrocellulose membranes (Scheicher & Schuel, Dassel, Germany). Non-specific binding was blocked overnight with 20% non-fat milk and Tris-buffered saline, containing 0.01% Tween-20. The membranes were incubated for 1 h with 1:2000 primary antibody, washed and incubated for 1 h with appropriate horseradish peroxidase-conjugated secondary antibody, then detected by chemiluminescence (ECL; Beit-Ha'Emek, Israel). The bands were quantified using a densitometer system endowed with BioCapt and TINA software (Raytest, Staubenhardt, Germany). Primary antibodies used: Ets-1, Ets-2, Elk-1, Net, PEA3, Erg 1/2/3, C/EBP α, C/EBP β, C/EBP γ, C/EBP λ, C/EBP ε, p53, phospho-p53 (Ser 392 phosphorylated p53), AP-2α, Ap-2β, AP-2γ (Santa Cruz, Biotech, California, USA).

### Transient transfection of antisense against Ets-2 or p53

JAR cells (5 × 10^5 ^per well) were plated on 24-well plates and transfected in duplicates with 750 nmol/liter antisense against humanEts2 (5'-/FAM/TTC CTT CCC ACC CTC CTA CC-3') or p53 (5'-/FAM/TCC GTC CCA GTA GAT TAC CAC-3) (IDT Inc., Hy-labs, Israel) in Optimem medium (Gibco, Invitrogen, Dorset, UK) using Lipofectamine (Invitrogen, Dorset, UK), according to manufacturer's protocol. After 6 hours, media with 20% serum was added to transfection media. After 24 hours post-transfection, cells were incubated in 10% FCS for 6 h and then plated for the various experiments. A reduction of at least 50% in Ets-2 or p53 distribution was considered a successful transfection.

### Immuno-fluorescence staining

JAR cells or 1^st ^trimester trophoblasts were cultured on glass coverslips in 35 μl medium drops under mineral oil. Cells were washed with PBS and fixed with 3.7% Paraformaldehyde (Electron Microscope Sciences, Belgar) in PBS for 10 minutes at 4°C, then washed twice with PBS and permeabilized for 5 minutes at 4°C with 0.1% Triton (Sigma, St. Louis, MO, USA) in PBS. Slides were incubated for 1 hour with blocking buffer 3% BSA-PBS), washed 3 times with PBS and incubated for 30 minutes at room temperature with 1 μg primary antibodies (Anti MMP-2, Anti Ets-2, Anti p53). Slides were rinsed five times with PBS, then incubated for 30 minutes with 0.5 μg secondary antibody (for F-actin:phalloidin, AlexaFlour-488, A-12379; for Ets-2 and p53: goat anti-rabbit IgG conjugated with AlexaFlour-546; for MMP-2: goat-anti mouse IgG conjugated with AlexaFlour-633, all from Molecular Probes, Invitrogen, Dorset, UK). Stained cells were photographed using a confocal microscope (Bio-Rad radiance 2000 confocal set-up with a fluorescent microscope Nikon E600 with a 60× lens). The photos were analyzed by Image Pro software (Media Cybernetics, Bethesda, USA), which quantifies density per area.

### Statistical methods

All data are expressed as mean ± SEM. Statistical analysis of the data was performed using Student's t-test. P < 0.05 was considered significant.

## Results

### The effect of forskolin on MMP-2 expression in JAR and 6-8 w trophoblast

**Figure 1:**
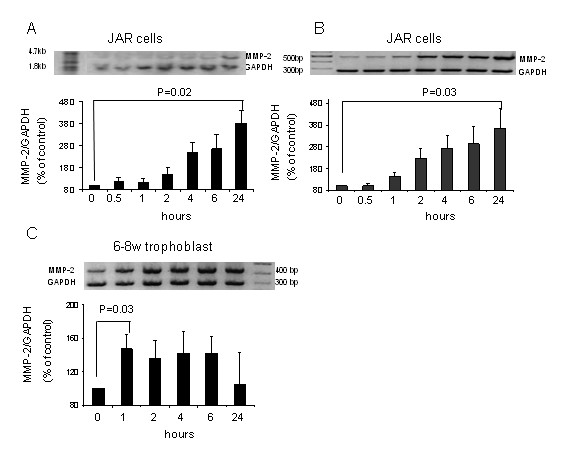
**Effect of forskolin on MMP-2 expression in JAR and 6-8 w trophoblast**. *Top panel*, total RNA of MMP-2 isolated form JAR (**A+B**) or 6-8 w trophoblast (**C**) cultured with Forskolin (10 μM) for the indicated times was assessed by Northern blot (**A**) and semiquantitative reverse transcription-PCR (**B+C)**. *Bottom panel*, band intensities were quantified with Densitometer system endowed with TINA software and the data presented as the ratio of MMP-2 to GAPDH (percent of control). The value represents the mean ± SEM from six different experiments performed in duplicates.

In order to explore the effect of Forskolin upon MMP-2 expression in JAR cells both Northern blot and RT-PCR was performed using the same RNA samples. In order to compare the results of the cell-line with primary cells RT-PCR was performed with 6-8 w 1^st ^trimester trophoblast. Forskolin was added to JAR or 6-8 w trophoblast culture for various length of time, in order to find the optimal exposure. Figure 1 summarizes the results. The mRNA of MMP-2 was increased in JAR cells by Forskolin (10 μM), reaching a maximum after 24 hours of incubation (382% ± 60, P = 0.02, Fig. 1A, 363% ± 90, P = 0.03, Fig. 1B). In 6-8 w trophoblast a maximum was reached within 1 hour after adding Forskolin (147% ± 17, P = 0.03, Fig. 1C). These exposures were chosen for further studies as optimal exposure for MMP-2 induction.

### Effect of forskolin on JAR nuclear protein binding to oligonucleotides sequences resembling binding sites found in the MMP-2 promoter

**Figure 2:**
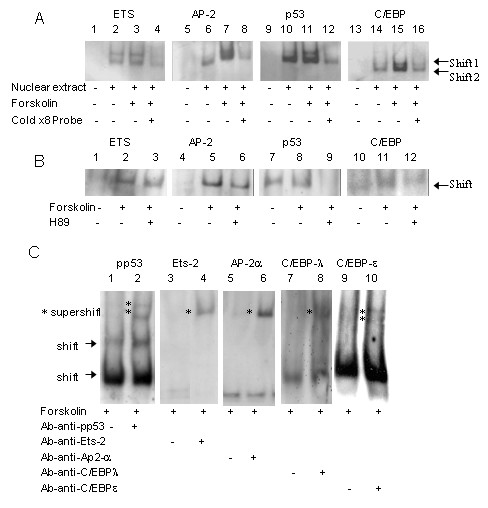
**Effect of forskolin on JAR nuclear protein binding to oligonucleotides corresponding to MMP-2 promoter binding sites**. **A**, binding of nuclear proteins, extracted from JAR cells cultured with or without Forskolin (10 μM), to dig-labeled oligonucleotides corresponding to consensus binding sites in the MMP-2 promoter was assessed with EMSA. The specificity of the DNA-protein complexes was tested by competing with a 8-fold excess unlabeled probe (lane 4, 8, 12, 16) The pictures are representative results of twelve different experiments. **B**, binding of nuclear proteins extracted from JAR cells treated with or without Forskolin (10 μM) and in the presence or absence of PKA inhibitor H89 to dig-labeled oligonucleotides was assessed with EMSA. The pictures are representative results of five different experiments. **C**, Specific antibodies against relevant transcription factors were added to the reaction mixture and EMSA supershift assay performed to detect transcription factors binding to the complex of dig-labeled oligonucleotides and nuclear proteins from JAR cells cultured with 10 μM Forskolin. **Lane 1-2**, addition of phosho-p53 (pp53) antibody to cell extract. **Lane 3-4**, Addition of Ets-2 antibody. **Lane 5-6**, Addition of Ap-2α antibody. **Lane 7-8**, Addition of C/EBPλ antibody and **lane 9-10, **addition of C/EBPε antibody to cells treated with Forskolin. Supershifted bands are designated with an asterisk (*). This result is representative of seven different experiments.

In order to detect transcription factors possibly involved in cAMP induction of MMP-2 in trophoblasts, we performed Gelshift assay (EMSA) of nuclear extract obtained from cells cultured for 24 hours in the presence or absence of 10 μM Forskolin. The binding of nuclear proteins to labeled oligonucleotides (Table 1), with sequences resembling binding sites found in the MMP-2 promoter, was explored. This method has been commonly used to detect possible binding sites and factors involved in transcriptional regulation [27-29]; Figure 2 summarizes the results. Forskolin increased binding to ETS, C/EBP, AP-2 and p53 DNA binding sites (Fig. 2A). No increase was found in binding to oligonucleotides SP-1, NF_k_B, AP-1 and CREB (data not shown). In order to assure binding specificity, competing non-labeled 8x excess oligonucleotide was added to reaction mixture. The non-labeled excess oligonucleotide strongly reduced the gelshift band, hereby ensuring binding specificity. Additionally, in order to assure involvement of the PKA signaling pathway, specific PKA inhibitor H89 (100 μm) was added to cell culture and gelshift performed (Fig 2B). H89 reduced binding activity and thereby confirmed the involvement of the PKA pathway (lane 3, 6, 9, 12).

### Identification of transcription factors binding to oligonucleotides involved in forskolin- induced MMP-2 expression

In order to explore transcription factors involved in Forskolin-induced MMP-2 expression, supershift EMSA was performed. In this assay, antibodies that recognize and identify the specific protein, bind to the protein-oligonucleotide complex, resulting in a higher, heavier band. Specific antibodies belonging to the families of transcription factors capable of binding to the relevant oligonucleotides were added to nuclear extract, prior to the binding reaction with oligonucleotides. Figure 2C shows the results. Antibodies to phospho- p53 (lane 2), to Ets-2 (lane 4), to Ap-2α (lane 6) and to C/EBPλ (lane 8) and C/EBPε (lane 10) supershifted the EMSA complex in Forskolin-treated cells. No supershift was found with other members of the various transcription factor families: ETS-1, Elk-1, Net, PEA3, Erg 1/2/3 (ETS); C/EBPα, c/EBPβ, C/EBPγ (C/EBP); p53 (p53); AP-2β, AP-2γ (AP-2) (data not shown).

#### Detection of transcription factors induced by forskolin and possibly involved in MMP-2 regulation

**Figure 3:**
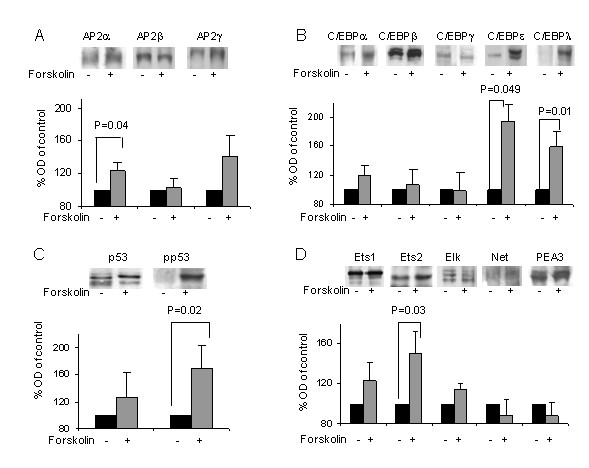
**Detection of transcription factors induced by forskolin in JAR cells**. The expression of various members of transcription factor families, capable of binding to AP-2, p53, C/EBP and ETS binding sites, found in the MMP-2 promoter, was examined with western blot. JAR cells were cultured in the absence or presence of 10 μM Forskolin and total nuclear proteins extracted. Band intensities were quantified with Densitometer system endowed with TINA software and the data represents the mean ± SEM from 10 different experiments. *Top panel *shows representative pictures. *Bottom panel *- graphs showing quantification of bands. The control band intensity was indicated as 100 percent and is represented by black square. Gray square represents nuclear extract from Forskolin treated cells. **A**, AP-2 family; **B**, C/EBP family; **C**, p53 family; **D**, Ets family.

**Figure 4:**
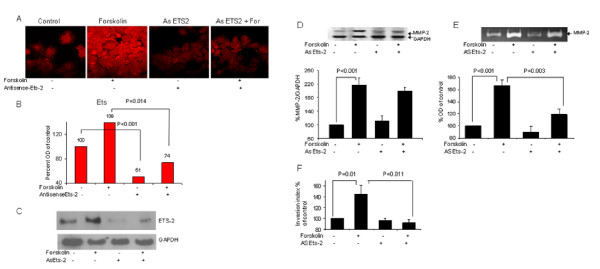
**Inhibitory effect of Ets-2 antisense upon forskolin-induced MMP-2 expression and secretion and trophoblast invasiveness**. **A**, JAR cells were transfected with antisense against Ets-2 (As Ets-2), then cultured in the presence or absence of 10 μM Forskolin, simultaneously with non-transfected cells, then fixed and stained with a fluorescent antibody against Ets-2 (**A**), or nuclear proteins extracted and western blot performed (**C**). The results are representative photos taking with a confocal microscope, magnification × 60. **A-C **Results represent 3 different experiments performed in duplicates. **B**, Density of ETS-2 staining was quantified with Image software and presented by Bar graph. **C**, representative pictures of western blot. **D**, *Top panel*, Total RNA of MMP-2 from JAR cell, with or without treatment with Ets-2 antisense and in the presence or absence of Forskolin was assessed by semi-quantitative reverse transcription-PCR. **D**, *Bottom panel*, band intensities were quantified with Densitometer system and presented as ratio of MMP-2 to GAPDH, as percent of control. Data represents mean ± SEM from 3 independent experiments performed in quadruples. **E**, MMP-2 secretion (72 kD) was assessed by zymography of conditioned media. **E ***Top *panel: Representative zymography gels. **E**, *Bottom panel*, Bar graph, representing mean ± SEM from 3 independent experiments performed in quadruplets. The control band intensity was indicated as 100 percent. **F**, Bar graph representing cell invasion ability of transfected and non-transfected JAR cells tested with Transwell Invasion Assay from 4 different experiments performed in duplicates.

EMSA can only show which transcription factors perform increased binding to their respective binding sequence under stimulation of Forskolin but not whether these factors indeed bind to the MMP-2 promoter. In order to further verify our results we performed a complementary study, using western blot to detect transcription factors increased in the nucleus by Forskolin. Nuclear cell extract was prepared from JAR cells cultured 24 hours in the presence or absence of 10 μM Forskolin and western blot performed with antibodies against the same transcription factors previous tested with EMSA. Figure 3 shows the results. Forskolin increased significantly nuclear expression of AP-2 by 123% ± 11, P = 0.043 (Fig. 3A), of C/EBPε by 150% ± 24, P = 0.049 (Fig. 4B), of C/EBPλ by 159% ± 22, P = 0.007 (Fig. 3B), of phospho-p53 by 169% ± 34, P = 0.021 (Fig. 3C) and of Ets-2 by 151% ± 22, P = 0.03 (Fig. 3D).

Of the 5 different transcription factors Ets-2, phospho-p53, Ap-2α, C/EBPε and C/EBPγ, shown to be increased by Forskolin, Ets-2 and p53 were chosen for further study of their contribution to MMP-2 transcriptional regulation in trophoblasts. Both Ets-2 and p53 have previously been suggested to be involved in the implantation process [30-34].

### Involvement of Ets-2 in forskolin- induced MMP-2 expression and trophoblast invasiveness

In order to further examine the involvement of Ets-2 in Forskolin- induced MMP-2 transcriptional regulation, we performed a silencing study using antisense against Ets-2. Ets-2 was inhibited by transfection of JAR cells with a specific antisense against Ets-2. Cells were then cultured in the absence or presence of Forskolin and four different parameters were analyzed. Figure 4A-F shows the results. Fluorescent antibody staining against Ets-2 and western blot analysis confirmed the inhibitory effect of the antisense. The transient transfection with Ets-2 antisense caused a 49% decrease in Ets-2 expression in non treated cells (100% vs.51% ± 8, P < 0.05), and a 65% decrease in Forskolin-treated cells (139% vs. 74% ± 6, P < 0.05) (Fig. 4A, B, C).

The inhibition of Ets-2 did not significantly affect the Forskolin-induced expression of MMP-2 as assessed by RT-PCR (216% vs.200% ± 10, not significantly) (Fig. 4D). However, a significantly 47% decrease in MMP-2 secretion in transfected cells, compared with non-transfected cells, both Forskolin-treated, was found by zymography (166% vs. 119% ± 9, P < 0.05) (Fig. 4E). In addition Ets-2 inhibition caused a 52% decrease in trophoblast invasiveness, compared with non-transfected cells, both cultured with Forskolin (144% vs. 92% ± 6, P < 0.05) (Fig. 4F).

### Involvement of p53 in forskolin- induced MMP-2 expression and trophoblast invasiveness

**Figure 5:**
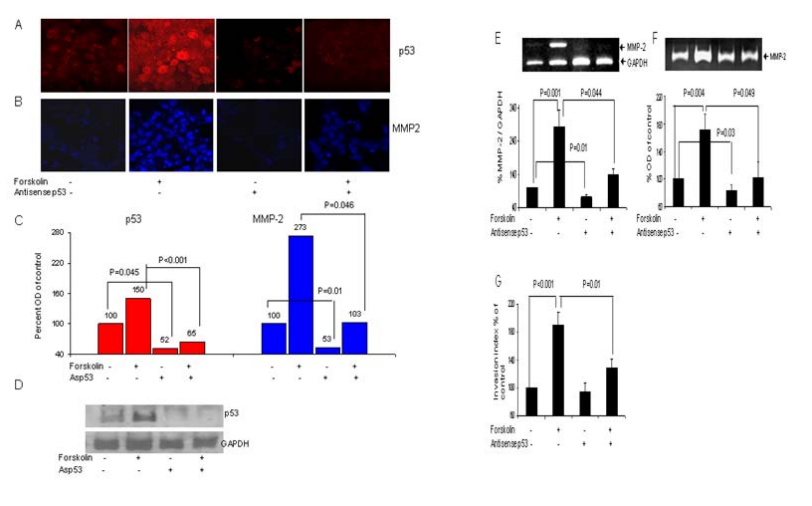
**Inhibitory effect of p53 antisense upon forskolin-induced MMP-2 expression and secretion and trophoblast invasiveness**. JAR cells were transfected with antisense against p53, then cultured in the presence or absence of 10 μM Forskolin, simultaneously with non-transfected cells. **A**,**B**, Representative photos taking with a confocal microscope of JAR cells, magnification × 60. JAR cells were fixed and stained with a fluoroscent antibody against p53 (**A**) or against MMP-2 (**B**), or nuclear proteins extracted and western blot performed (**D**). **A-D **Results represent 3 different experiments performed in duplicates. **C, **Density of p53 and MMP-2 staining was quantified with Image software and presented by Bar graph, black-p53, grey-MMP-2.**D, **representative pictures of western blot.**E***Top panel*, Total RNA of MMP-2 from JAR cell, with or without treatment with Ets-2 antisense and in the presence or absence of Forskolin was assessed by semiquantative reverse transcription-PCR.**E**, *Bottom panel*, band intensities were quantified with Densitometer system and presented as ratio of MMP-2 to GAPDH, as percent of control. Data represents mean ± SEM from 4 independent experiments performed in duplicates. **F**, MMP-2 secretion (72 kD) was assessed by zymography of conditioned media. **F**, *Top *panel: Representative zymography gels. **F**, *Bottom panel*, Bar graph, representing mean ± SEM from 3 independent experiments performed in quadruplets. The control band intensity was indicated as 100 percent. **G**, Bar graph representing cell invasion ability of transfected and non-transfected JAR cells tested with Transwell Invasion Assay from 3 different experiments performed in quadruplets.

JAR cell were transfected with specific antisense against p53, cultured in the presence or absence of Forskolin and experiments of four parameters performed. Figure 5 shows the result. The transient transfection with p53 antisense decreased p53 expression by 48% in control cells (100% vs. 52% ± 11, P < 0.05) and by 85% in Forskolin-treated cells (150% vs. 65% ± 18, P < 0.05) (Fig. 5A, C). The inhibition of p53 decreased the Forskolin-induced expression of MMP-2 by 85%, compared to non-transfected cells, as assessed with immunofluorescent staining (273% vs. 103% ± 37, P < 0.05) (Fig. 5B, C) and by 146% as assessed with RT-PCR (283% vs. 137% ± 21, P < 0.05) (Fig. 5E). This result shows that p53 transcription factor is involved in MMP-2 transregulation by Forskolin. p53 inhibition also reduced MMP-2 expression in control cells without Forskolin by 29% (RT-PCR) (100% vs. 71% ± 10, P < 0.05) or by 47% (immunostaining) (100% vs. 53% ± 15, P < 0.05) (Fig. 5E, C). This indicates that the p53 transcription factor is also involved in the constitutive expression of MMP-2. A correspondingly decrease in MMP-2 secretion was found by zymography in both groups (Fig. 5F): a 69% decrease in the presence of Forskolin (171% vs. 102 ± 23, P < 0.05) and a 18% decrease without Forskolin (100% vs. 82% ± 9, P < 0.05). In addition, p53 inhibition caused a 61% decrease in trophoblast invasiveness, compared with non-transfected cells, both cultured with Forskolin (190% vs. 129% ± 12, P < 0.05) (Fig. 5G).

### The effect of forskolin on Ets-2 and p53 in 6-8 w 1st trimester trophoblasts - verification of similarity with cell-line results

**Figure 6:**
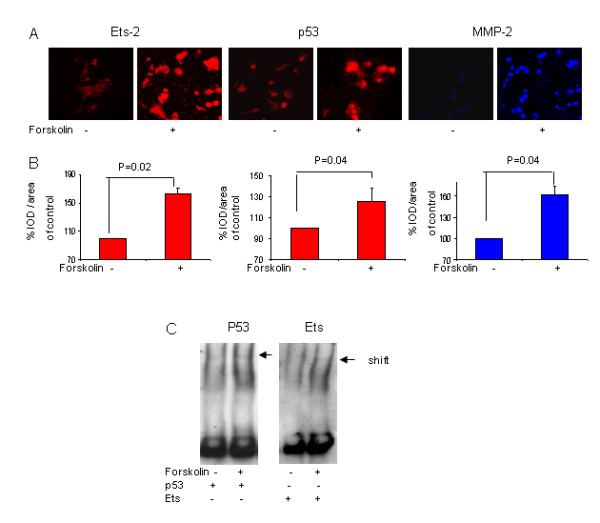
**The effect of forskolin on Ets-2 and p53 expression in 6-8 w 1^st ^trimester trophoblasts**. **A**, Representative photos taking with a confocal microscope of 6-8 w 1^st ^trimester trophoblast, magnification × 60. 6-8 w 1^st ^trimester trophoblast, cultured with or without Forskolin (10 μM), were fixed and stained with a fluorescent antibody against Ets-2, p53 or MMP-2. **B**, Density of p53, Ets-2 and MMP-2 staining was quantified with Image software and presented by Bar graph, **left**-Ets-2, **middle**-p53, **right**-MMP-2. **C, **Binding of nuclear proteins extracted from 6-8 w 1^st ^trimester trophoblast cells treated with or without Forskolin (10 μM) to dig-labeled oligonucleotides Ets and p53 was assessed with EMSA. Results represent 2 different experiments performed in triplicates.

Trophoblast cell-lines are excellent models for studying the mechanism of implantation, but results should always be verified with primary cells. We therefore performed EMSA and immunofluorescent staining of Forskolin-treated 6-8 w 1^st ^trimester trophoblastic cells in order to verify the presence and involvement of Ets-2 and p53 in these primary cells. 6-8 w 1^st ^trimester trophoblast were cultured in the presence or absence of 10 μM Forskolin, then cells were fixed and stained with fluorescent antibodies against Ets-2, p53 and MMP-2, or total nuclear protein was extracted and EMSA performed with oligonucleotides Ets and p53. Figure 6 summarizes the results. Forskolin increased Ets-2, p53 and MMP-2 expression significantly by 164 ± 7, 125 ± 12 and 162 ± 13, respectively (Fig. 6A, B), and increased binding activity to oligonucleotides p53 and Ets in 6-8 w 1^st ^trimester trophoblasts, like previously found with JAR cells (Fig. 6C). This confirms the resemblance between 6-8 w 1^st ^trimester trophoblast and JAR cells and indicates that the results obtained with JAR cell-line may be assumed to apply to early trophoblast with regards of gelatinolytic activity, regulation and invasiveness.

## Discussion

We have found that Forskolin increases both trophoblast MMP-2 activity and MMP-2 mRNA expression. The maximal mRNA response in JAR cells appeared only after 24 hours of incubation, suggesting that Forskolin may stimulate MMP-2 by inducing other, early response genes. The MMP-9 gene has similarly been reported to be induced within 24 h in trophoblasts by 12-O-tetradecanoyl phorbol 13-acetate (TPA) and TNF, through transcription of early response genes [29]. In 6-8 w trophoblasts, in contrast, Forskolin induced MMP-2 mRNA within 1 h, indicating that Forskolin apparently can stimulate MMP-2 expression more directly, probably through initiating phosphorylation and migration of already present transcription factors. The controversy found here between JAR cells and 6-8 w trophoblasts, in regards of time for maximal mRNA response to Forskolin, underlines that some differences are to be expected between primary cells and a cell-line. One can speculate that in choriocarcinoma cells like JAR, an altered presence of transcription factors or activation of phosphorylation cascades may be part of the malignancy of these cells. The difference observed in ultimate magnitude between JAR cells and 1^st ^trimester trophoblasts in response to Forskolin is consistent with our previous findings, showing the invasive choriocarcinoma cell-line to be highly expressing MMP-2, both in response to Forskolin and EGF [8]. The primary trophoblast are less invasive and express less MMP-2 than the JAR cells.

Forskolin increased the binding of nuclear proteins to oligonucleotides corresponding to the consensus sequence of AP-2, C/EBP, p53 and Ets response elements in the MMP-2 promoter. These binding activities were induced specifically through PKA, since specific PKA- inhibitor H89 reduced this effect. The supershift EMSA assay revealed, that transcription factors AP-2α, phospho- p53, Ets-2 and C/EBPε and -λ bind to their respective binding sequence under stimulation of Forskolin. Forskolin also increased the protein expression of Ets-2, C/EBPε, C/EBPλ and AP-2α, and the phosphorylation of p53 as shown by western blot. These transcription factors may therefore be involved in transregulation of the MMP-2 promoter by Forskolin.

Several transcription factors have been found to be involved in MMP-2 regulation in various cells, including AP-2, AP-1 and p53 [28,35,36]. Only a few studies of MMP-2 transcriptional regulation in trophoblast have been published, and have focused mainly on the signaling pathway involved. In 1^st ^trimester trophoblast the MAPK/Erk1/2 pathway was found to be involved in induction of MMP-2 and MMP-9 by Cyclosporin A [37]. A cytoplasmatic tyrosine kinase named focal adhesion kinase was found to be involved in cell differentiation, adhesion, proliferation and MMP-2 activity in early trophoblast [38]. In another study from our lab, progesterone was found to regulate MMP-2 through differences in progesterone-receptor isoforms, apparently by binding to co-activators or transcription factors, since there is no progesterone response element in the MMP-2 promoter [39]. hCG, which like Forskolin acts through PKA, induces MMP-2 expression and trophoblast invasion through ERK 1/2 and PI3K/AKT pathways [40]. Our study is the first to focus on transcription factors, which mediate MMP-2 transcriptional regulation in trophoblasts. We chose to focus our further investigation upon two transcription factors, Ets-2 and p53, which have previously been suggested to be involved in the implantation process. We showed that that Ets-2 mediates Forskolin-induced MMP-2 activity, since Forskolin increased Ets-2 expression in trophoblast and since antisense inhibition of Ets-2 reduced the Forskolin-induced MMP-2 gelatinolytic activity and trophoblast invasion. Inhibition of Ets-2 did not significantly reduce MMP-2 transcription, indicating that this transcription factor alone, although essential to MMP-2 activity and function, is not sufficient to MMP-2 transcriptional regulation. Based on our results we hypothesize that Ets-2 may effect MMP-2 regulation on a post-transcriptional level, however further investigation is necessary to elucidate the exact mechanism.

The ETS transcription factor family is characterized by an highly evolutionary- conserved DNA- binding domain, that binds to a purine- rich GGA(A/T) core sequence [41,42]. Ets members direct cytoplasmic signals to control gene expression and mediate multiple signaling pathways such as the Ras- Map Kinases, Erk1,2, p38, JNK and the PI3 kinases, which control their activity, protein partnership and specification of downstream target genes [41-43]. Ets members have been shown to interact with several transcription factors including AP-1, pit-1, NF_k_B and SP-1 [34,42,44]. Ets proteins play important roles in cell development, differentiation and proliferation, and participate in malignancy of tumor cells, including invasion and metastasis, by activating the transcription of several cancer-related genes such as proteases and angiogenesis-related genes [41]. Binding sites for the ETS family are found in promoters of the MMP family [45], and regulation of matrix degrading proteases by Ets factors in tumor invasion and metastasis is well established [46]. Ets-2 is expressed in many cell types in developing mouse embryos. Targeted deletion of Ets-2 in mice results in retardation and death of the mouse embryo before 8.5 days of development [34]. These embryos show defects in the extraembryonic tissue gene expression and function, including deficient expression of MMP-9, MMP-13 and MMP-3 and die as a result of trophoblast failure [34,47]. Apparently. Ets-2 is not essential for specifying trophoectoderm before the morula-blastocyst transition, since blastocyst do form and initiate implantation. Properly Ets-2 coordinates early expression of genes providing full characteristics to cytotrophoblast, including the production of key enzymes and hormones [47]. MMP-2 may be one of these genes. Ets proteins mediate the control of several genes characteristically up-regulated in human trophoblast and involved in trophoblast differentiation and placenta development [32]. This includes the hCG-α and hCG-β subunit gene, placental lactogen II, p-450 side chain cleavage enzyme, decidual/trophoblast prolactin related gene and MMP-1, -3 [48-51]. The hCG subunits are activated in stimulation to cAMP through interactions between Ets and CREB [32,47].

We also found that Forskolin increases p53 expression, and that p53 mediates Forskolin-induced MMP-2 expression, gelatinolytic activity and both basic and PKA-induced trophoblast invasion. In contrast to Ets-2, transient transfection of antisense mRNA to p53 into trophoblasts decreased MMP-2 transcription, both in control and in Forskolin-treated cells. This indicates, that p53 may be involved in the constitutive expression of MMP-2, and additionally mediates the response to PKA. We have recently published that p53 is involved in MMP-2 regulation and trophoblast invasion by EGF [52]. p53 was among the transcription factors increased under EGF stimulation (p53, SP-1, AP-2α and -γ, C/EBPε and-λ). p53 antisense inhibition was found to decrease EGF- induced MMP-2 mRNA expression, secretion and trophoblast invasiveness. Together our two studies emphasize the conclusion that p53 is involved in MMP-2 regulation in trophoblast. Since AP-2α and C/EBPε and -λ were also increased by both stimulators, they should be further examined by functional assays. Other transcription factors, like ETS-2, may be specific for a certain pathway.

p53 is a 53 kDa nuclear transcription factor, that recognizes and binds to a specific DNA consensus sequence consisting of two copies of the 10 bp motif 5'-PuPuPuC(A/T)(T/A)GPyPyPy-3', separated by 0 to 13 bp [53]. p53 acts as an activator by binding to this sequence and can also act as a transcriptional repressor of promoters lacking a p53 binding site [54-56]. p53 activates genes involved in DNA repair [30]. Biologically p53 induces G1 arrest and apoptosis following DNA damage [57], and is therefore known as a common tumor suppressor [58]. Many p53 functions are mediated through the activation or repression of a series of genes, and this includes the MMP-2 gene [36]. Mutation of p53 may inactivate the growth regulatory function and cause a loss of tumor-suppressive activity [59]. Mutation at the specific DNA binding domain have been identified in nearly 50% of all human cancers [60]. p53 is present in the preimplantation human embryo [61,62], and is expressed throughout mouse implantation development. The p38 MAPK pathway was found to regulate p53 expression in 2-8 cell mouse embryos [63]. p53-deficient mice are viable and reproductive, however in p53- null (p53(-/-) the placenta is altered [64]. In rodent placenta, a decrease in p53 is part of the transition of trophoblast from a state of proliferation to differentiation in trophoblast [33]. p53 has a short half-live, and is therefore normally present at very low concentrations in cells [30]. However, p53 is detectable in the nucleus and cytoplasm of cytotrophoblasts and syncytiotrophoblasts [30,65,66], and this apparently overexpression of p53 in trophoblasts may be controlling excessive proliferation in normal placentation [67]. It appears that most p53 protein forms complexes in the cytoplasm and tetramers in the nuclei in cytotrophoblasts, suggested to stabilize p53 and hereby prolong the half-life [30,31]. p53 is correlated with enhanced apoptosis in trophoblast [68], and can be induced by hypoxia [69]. Increased p53 and apoptosis was found in different pregnancy complications, such as preeclampsia [69,70], fetal growth restriction [68] and Gestational Trophoblastic diseases (GTDs) [71]. In invasive hydatidiform moles, characterized by abnormal growth of chorionic tissues with various degrees of local invasion and metastasis, MMP-2 and mutant p53, but not wild type p53 (wtp53), were increased, as compared to normal placenta, and may be involved in this pathology [72]. However, since mutant p53 may still perform normal suppressive activity, depending on the localization of the mutation, and since other reports found wtp53 to be elevated in hydatidiform moles [73-75], the role of wtp53 in this pathology is not clear. A recent article described that exogenous p53, but not endogenous p53, decrease MMP-9 in cytotrophoblasts [76]. In their report, in contrast to our results, p53 inhibition had no effect on either MMP-2 or MMP-9, and the authors suggested that p53 is inactivated in trophoblasts, maybe through formation of high molecular weight complexes. These controversial results regarding MMP-2 may result from differences in experimental design, including the use of JAR cells as a model as well as primary trophoblast of different gestational ages (early versus late 1^st ^trimester trophoblast).

The examined transcription factors in this study are involved in transactivation and transrepression of many genes, thus the EMSA does not allow us to conclude, that MMP-2 is one of them. However, the studies using transfection of p53 and Ets-2 antisense in JAR demonstrate that at least p53 is involved in Forskolin-induced MMP-2 expression, while Ets-2 may effect post-transcriptional regulation. Our results suggest that MMP-2 regulation in trophoblast is mediated by several transcription factors, including p53 and Ets-2, however since MMP-2 transcription, secretion and trophoblast invasion were not completely diminished, other transcription factors must be involved. This may include C/EBP-ε, C/EBPλ and AP-2α, which should be subject to further study.

## Conclusion

MMP-2 regulation by PKA is mediated through several binding sites and transcription factors including Ets-2, p53, C/EBPε, C/EBPλ and AP-2α. Ets-2 and p53 mediate PKA- induced trophoblast invasiveness through regulation of MMP-2 expression and activity.

## Competing interests

The authors declare that they have no competing interests.

## Authors' contributions

ESR participated in designing the study, carried out the experiments, performed the data analysis and drafted the manuscript. SG participated in conceiving and designing the study, and helped with the data analysis and with drafting the manuscript. ES conceived and design the study, analyzed the results and edited the manuscript. All authors read and approved the final manuscript.
